# Geranylgeraniol Inhibits Lipopolysaccharide-Induced Inflammation in Mouse-Derived MG6 Microglial Cells via NF-κB Signaling Modulation

**DOI:** 10.3390/ijms221910543

**Published:** 2021-09-29

**Authors:** Wahyu Dwi Saputra, Hiroki Shono, Yusuke Ohsaki, Halima Sultana, Michio Komai, Hitoshi Shirakawa

**Affiliations:** 1 Laboratory of Nutrition, Graduate School of Agricultural Science, Tohoku University, 468-1 Aramaki Aza Aoba, Aoba-ku, Sendai 980-8572, Japan; wahyu@g-mail.tohoku-university.jp (W.D.S.); hiroki.shono.t5@dc.tohoku.ac.jp (H.S.); yusuke.ohsaki.a4@tohoku.ac.jp (Y.O.); sultana.halima.d4@tohoku.ac.jp (H.S.); mkomai@m.tohoku.ac.jp (M.K.); 2 International Education and Research Center for Food Agricultural Immunology, Graduate School of Agricultural Science, Tohoku University, 468-1 Aramaki Aza Aoba, Aoba-ku, Sendai 980-8572, Japan

**Keywords:** geranylgeraniol, microglial cells, inflammation, NF-κB

## Abstract

Persistent inflammatory reactions in microglial cells are strongly associated with neurodegenerative pathogenesis. Additionally, geranylgeraniol (GGOH), a plant-derived isoprenoid, has been found to improve inflammatory conditions in several animal models. It has also been observed that its chemical structure is similar to that of the side chain of menaquinone-4, which is a vitamin K_2_ sub-type that suppresses inflammation in mouse-derived microglial cells. In this study, we investigated whether GGOH has a similar anti-inflammatory effect in activated microglial cells. Particularly, mouse-derived MG6 cells pre-treated with GGOH were exposed to lipopolysaccharide (LPS). Thereafter, the mRNA levels of pro-inflammatory cytokines were determined via qRT-PCR, while protein expression levels, especially the expression of NF-κB signaling cascade-related proteins, were determined via Western blot analysis. The distribution of NF-κB p65 protein was also analyzed via fluorescence microscopy. Thus, it was observed that GGOH dose-dependently suppressed the LPS-induced increase in the mRNA levels of *Il-1β*, *Tnf-α*, *Il-6*, and *Cox-2*. Furthermore, GGOH inhibited the phosphorylation of TAK1, IKKα/β, and NF-κB p65 proteins as well as NF-κB nuclear translocation induced by LPS while maintaining IκBα expression. We showed that GGOH, similar to menaquinone-4, could alleviate LPS-induced microglial inflammation by targeting the NF-kB signaling pathway.

## 1. Introduction

Microglia are brain-resident macrophage cells that act as the first immune defense in the brain and are responsible for maintaining central nervous system (CNS) homeostasis conditions [1,2]. They are sensitive cells that can be transformed to have distinct characteristics that could be either favorable or harmful, depending on the duration and type of stimulus [3]. Upon activation by dangerous stimuli, such as brain injury or infection, microglia become active and produce various pro-inflammatory cytokines, including IL-1β, IL-6, and TNF-α [4]. When the stimulus is temporal, cytokine production can be controlled to return to the normal physiological state [5]. However, when such dangerous stimuli persist, the overproduction of inflammatory cytokines results in a cycle that further induces neuroinflammation [6,7]. This condition may cause neuronal cell loss, which can promote neurodegenerative pathogenesis. In a previous study, it was observed that the injection of a lipopolysaccharide (LPS), which a Gram-negative bacterial endotoxin, is capable of inducing microglial activation, which is often followed by neuron cell degradation in the animal brain, leading to cognitive impairment [8,9]. Moreover, elevated levels of inflammatory cytokines have also been confirmed in the cerebrospinal fluid of patients with neurological diseases [10,11].

Additionally, LPS binds to TLR4 in the cell membrane to activate inflammatory transcription factors, such as nuclear factor-κB (NF-κB) [12]. The essential mechanism associated with NF-κB activation is the phosphorylation and inducible degradation of IκBα protein, resulting in the nuclear internalization of NF-κB [13]. This IκBα event is triggered by the phosphorylation (activation) of its kinase, the IKK complex, which consists of IKKα and IKKβ as catalytic subunits, and NEMO as a regulatory subunit. IKK phosphorylation itself results from IRAK1-TRAF6 activation, which conveys signals via the TAK1 complex after LPS stimulation [14]. Furthermore, NF-κB modulates the expression of the mRNA of numerous genes that are involved in inflammatory response [15]. Therefore, targeting the NF-κB signaling pathway may be a beneficial therapeutic strategy for inhibiting inflammation-mediated disorders.

Geranylgeraniol (GGOH), which consists of four isoprene units, is a natural isoprenoid present in edible grains and vegetable oils [16,17,18]. Previous studies have revealed that its exogenous administration shows an anti-inflammatory effect in a mouse model of mevalonate kinase deficiency and inhibits NF-κB activation in hepatocarcinogenic rat models [19,20]. It has also been reported that GGOH inhibits the expression of LPS-induced pro-inflammatory markers in rats, macrophagic THP-1 cells, and amino bisphosphonates-treated RAW264.7 cells [21,22,23]. Furthermore, it has been suggested that GGOH is required to maintain endotoxin tolerance in peritoneal macrophages and counter IL-1β production in peripheral blood mononuclear cells from patients with mevalonate kinase deficiency [24,25]. These findings indicate that GGOH may modulate inflammation in the peripheral system.

**Figure 1:**
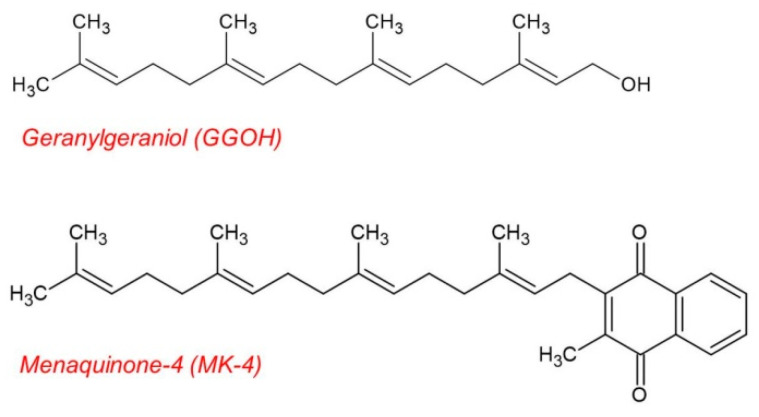
Chemical structure of geranylgeraniol (GGOH) and menaquinone-4 (MK-4). GGOH has a chemical structure that is similar to that of the side chain of MK-4, a vitamin K_2_ sub-type.

In CNS models, GGOH and geranylgeranyl-pyrophosphate (GGPP), a GGOH derivative, are reported to protect cultured neuron cells from the detrimental effects of statin administration [26,27]. In vitro experiments performed in previous studies have also indicated that low isoprenoid levels are associated with the accumulation of intracellular amyloid β (Aβ) [28,29]. Moreover, it has been observed that the suppression of geranylgeranyl products in statin-treated microglia triggers the upregulation of TNF-α [30]. Additionally, continuous GGOH synthesis in neuron cells is also suggested to be required for long-term potentiation and learning [31]. These findings suggest that isoprenoids, including GGOH, exert a positive effect in the CNS, especially in the regulation of mevalonate pathway (MVP). However, the function of GGOH, independent of MVP regulation in the CNS model, has not yet been adequately studied. Furthermore, the chemical structure of GGOH is similar to that of the side chain of menaquinone-4 (MK-4), a vitamin K_2_ sub-type, which we previously reported as showing the ability to inhibit NF-κB activation in MG6 cells (Figure 1) [32]. These facts prompted us to investigate whether GGOH possesses an anti-inflammatory effect that is similar to that of MK-4 in microglial cells.

Therefore, the aim of this study was to elucidate the anti-inflammatory effect of GGOH against LPS-induced inflammation in mouse-derived MG6 microglial cells, which is a confirmed cell line that is used as a microglial activation model [33,34]. By clarifying the molecular action of GGOH, we hope to lay a preliminary foundation for further investigation, especially regarding the association between GGOH and microglial cells.

## 2. Results

### 2.1. GGOH Inhibited the Upregulation of Pro-Inflammatory Cytokine Expression in MG6 Cells

**Figure 2:**
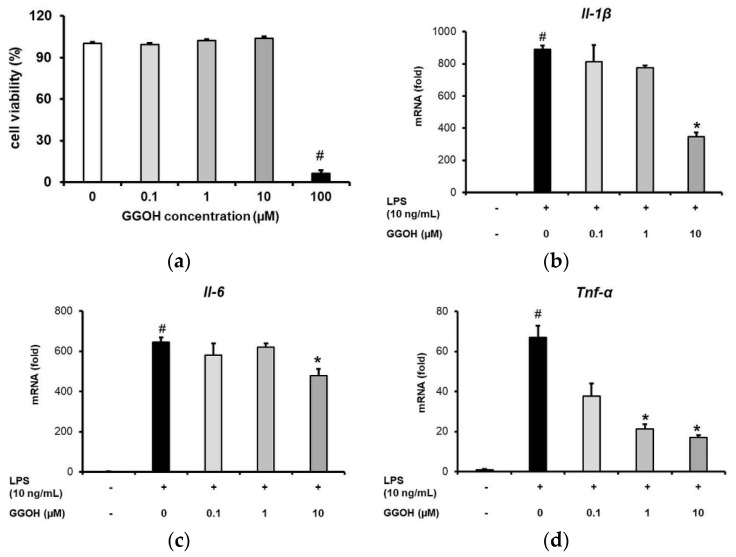

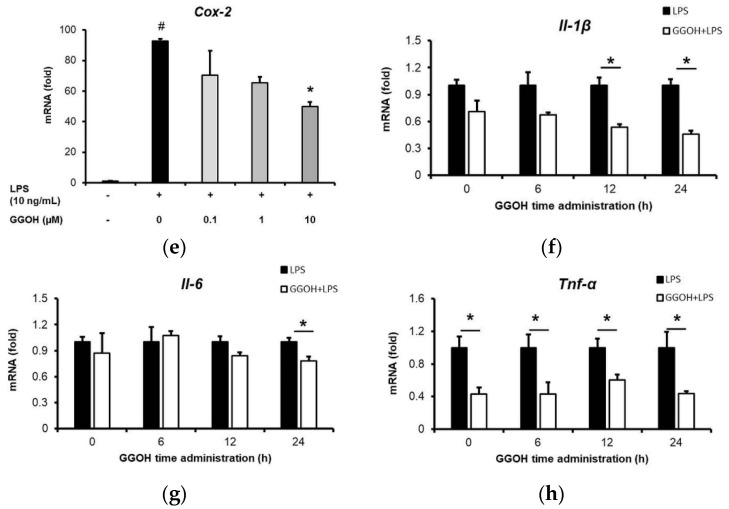
Suppression of pro-inflammatory cytokine mRNA expression by geranylgeraniol (GGOH). (**a**) MG6 cells pre-treated with GGOH at different concentrations for 24 h. Cell viabilities were evaluated using the WST-1 method. (**b**–**e**) Pro-inflammatory cytokine mRNA expression levels in MG6 cells pre-treated with GGOH at different concentrations for 24 h followed by LPS (10 ng/mL) treatment for 3 h. (**f**–**h**) Pro-inflammatory cytokine mRNA expression levels in MG6 cells pre-treated with GGOH (10 µM) at different times followed by LPS treatment (10 ng/mL) for 3 h. Closed bar, LPS-only treated groups; open bar, GGOH pre-treated groups. The mRNA levels were measured via quantitative reverse transcriptase-PCR, normalized to *Eef1a1* (the internal standard), and expressed as fold-changes relative to control cell values or the values corresponding to LPS-only treated MG6 cells. Data are presented as the mean ± S.E.M, n = 3; # *p* < 0.05 vs. untreated control; * *p* < 0.05 vs. LPS-only treated group).

First, an examination of the effect of GGOH on MG6 cell viability was performed. The results obtained revealed that GGOH administration at concentrations up to 10 µM did not affect the viability of the MG6 cells. However, a higher concentration (100 µM) markedly reduced the viability of the cells (Figure 2a). Hence, we set 10 µM as the maximum working dose in subsequent experiments. 

Pro-inflammatory cytokines are the major downstream products of inflammatory signaling. Here, we evaluated the anti-inflammatory action of GGOH by measuring pro-inflammatory cytokine mRNA expression levels resulting from the action of LPS following GGOH pre-treatment. As shown in Figure 2b–d, LPS administration for 3 h significantly upregulated the expression of interleukin-1β (*Il-1β*), interleukin-6 (*Il-6*), and tumor necrosis factor-α (*Tnf-α*) mRNA. However, pre-treatment with GGOH, especially at a concentration of 10 µM, markedly downregulated the mRNA expression levels of these pro-inflammatory cytokines by 2.5-, 1.3-, and 3.9-fold, respectively, compared with LPS-only treatment (Figure 2b–d). Furthermore, GGOH pre-treatment also inhibited the expression of cyclooxygenase-2 (*Cox-2*), which is a mediator of the inflammatory process (Figure 2e). Specifically, it effectively suppressed inflammatory cytokine expression after 24 h of administration, while its administration for less than 24 h had diverse effects on the expression levels of *Il-1β*, *Tnf-α*, and *Il-6* (Figure 2f–h). Therefore, in subsequent experiments, MG6 cells were incubated with GGOH for 24 h. The inhibition of pro-inflammatory transcriptional expression indicated that GGOH had an anti-inflammatory effect in LPS-induced inflammation in MG6 cells.

### 2.2. Mitigation of NF-κB Activation in MG6 Cells by GGOH

**Figure 3:**
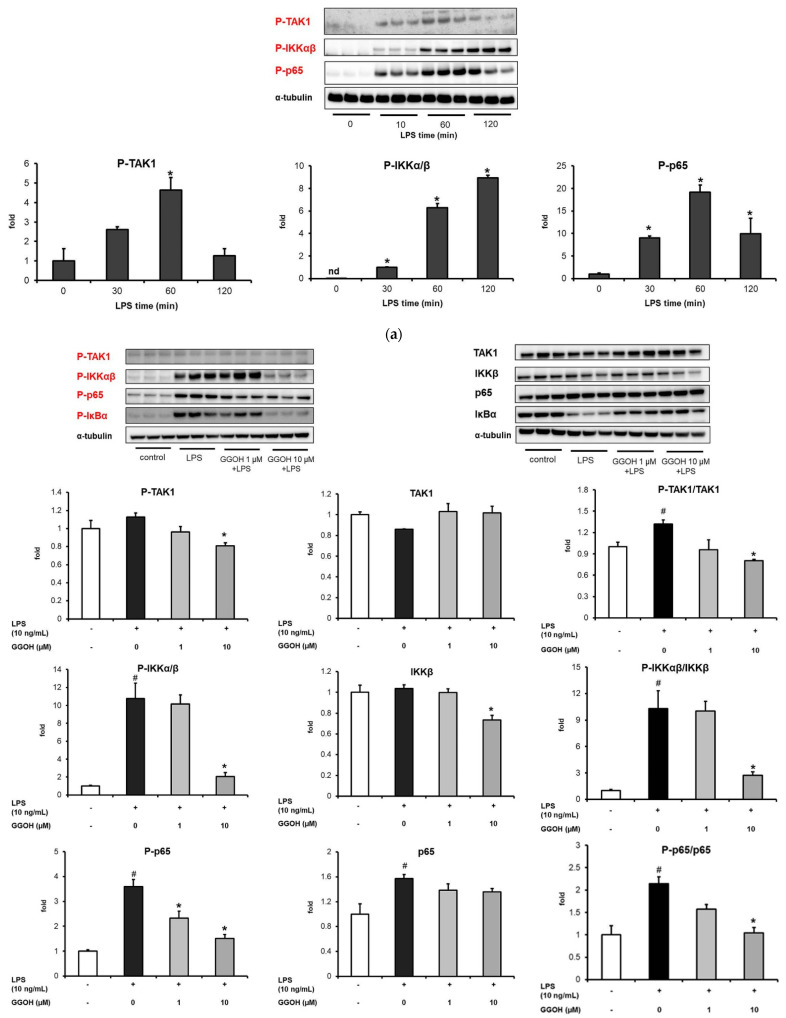

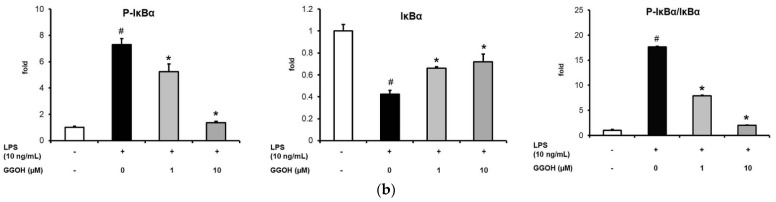
Inhibition of the expression of NF-κB-related proteins by geranylgeraniol (GGOH). MG6 cells were pre-treated with GGOH for 24 h followed by LPS treatment (10 ng/mL) for 1 h. (**a**) Western blot images and quantification of phosphorylated TAK1, IKKα/β, and the p65 subunit of the NF-κB signaling pathway after different LPS administration times. (**b**) Western blot images and quantification of phosphorylated and total TAK1, IKKα/β, p65, and IκBα levels. The ratio of phosphorylated to total TAK1, IKKα/β, p65, and IκBα proteins in the presence of GGOH pre-treatment is also shown. Total cell lysates were collected and subjected to Western blot analysis. Data are presented as the mean ± S.E.M, n = 3, normalized to the total protein levels detected based on α-tubulin. # *p* < 0.05 vs. untreated control; * *p* < 0.05 vs. LPS-only treated group or LPS-only at 0 min).

Transcription factors primarily drive the production of pro-inflammatory mediators [13,15]. Specifically, NF-κB is a transcriptional factor that is widely known to be triggered during LPS administration. Given that GGOH administration inhibited the expression of pro-inflammatory cytokines, we characterized the underlying molecular mechanism by assessing the expression of NF-κB signaling pathway-related proteins. First, the optimum LPS administration time required to induce NF-κB activation in MG6 cells was determined. As shown in Figure 3a, LPS induced the phosphorylation of TAK1, IKKα/β, and the p65 subunit of NF-κB after 30 min of administration. However, 1 h of LPS administration resulted in a more prominent result. Hence, 1 h was applied as the optimal time required for LPS administration in the Western blot experiments.

The GGOH pre-treatment group showed a significantly reduced capacity compared with the LPS-only treatment group with respect to TAK1, IKKα/β, and p65 phosphorylation based on their total protein levels (Figure 3b). We also examined the IκBα profile given that IκBα sequesters NF-κB in the cytoplasm in an inactive form. During activation, IκBα was phosphorylated and degraded by the proteasome, leading to the release of NF-κB. It was also observed that LPS triggered IκBα degradation in MG6 cells by 0.4-fold compared with the control group, while markedly enhancing IκBα phosphorylation. Conversely, GGOH notably inhibited IκBα degradation and phosphorylation, as shown in Figure 3b. These results indicated that GGOH arrested the NF-κB signaling pathway in LPS-induced inflammation in MG6 cells, contributing to the inhibition of pro-inflammatory cytokine expression.

### 2.3. Inhibition of the Nuclear Translocation of NF-κB by GGOH

**Figure 4:**
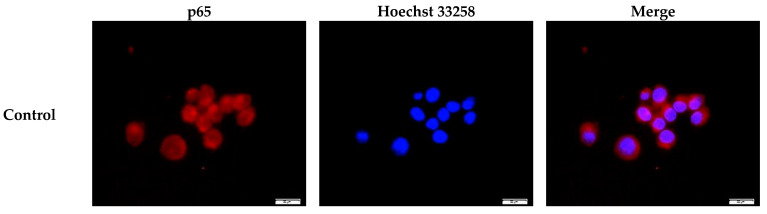

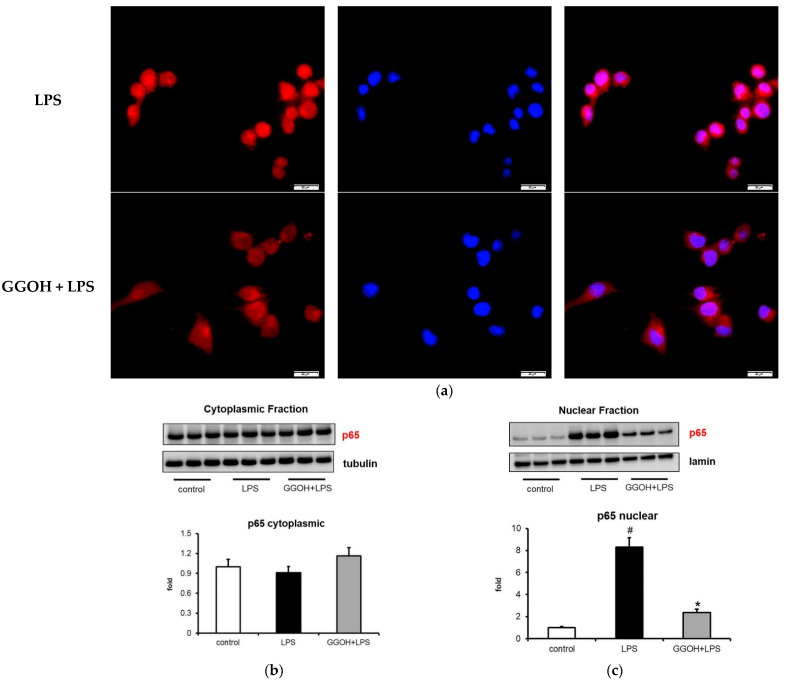
Inhibition of NF-κB p65 nuclear translocation by geranylgeraniol (GGOH) administration. MG6 cells were pre-treated with GGOH (10 µM) for 24 h followed by LPS treatment (10 ng/mL) for 1 h. (**a**) Cells were fixed and labeled with anti-p65 (red) antibodies. Nuclei were stained with Hoechst 33258 (1 μg/mL) (blue). Scale bars, 20 μm; (**b**) and (**c**) Subjection of the cytoplasmic and nuclear fractions of cells fractionated to Western blot analysis. Data are presented as the mean ± S.E.M, n = 3, normalized to total protein levels based on the use of α-tubulin or lamin, and expressed as the fold-change relative to the values corresponding to the control cells. # *p* < 0.05 vs. untreated control. * *p* < 0.05 vs. LPS-only treated group).

NF-κB activation is associated with nuclear translocation. Upon LPS administration, NF-κB enters the nucleus from the cytosol, binds to its DNA binding site, and activates pro-inflammatory downstream transcription [35]. Thus, we monitored whether GGOH also affects this phenomenon by evaluating the fluorescence distribution and cytoplasmic/nuclear blotting in the MG6 cells. Figure 4a shows representative images of the distribution of the p65 subunit of NF-κB p65 in MG6 cells. Under normal conditions, p65 was found to be primarily located in the cytoplasmic area (Figure 4a first panel). However, LPS triggered its nuclear translocation, as indicated by the intense p65 staining in the nuclear area (Figure 4a second panel, left and right photos). The latter phenomenon was reduced by GGOH pre-treatment (Figure 4a third panel). Next, this microscopic observation was verified by measuring NF-κB p65 levels in MG6 cytoplasmic and nuclear fractions. This analysis showed no significant variation in cytoplasmic p65 levels (Figure 4b). However, it was observed that LPS treatment significantly increased p65 levels by eight-fold in the nuclear fraction, while GGOH administration markedly mitigated this effect (Figure 4c). Hence, the results of our blotting experiment showed consistency with the microscopy observations, which indicated that GGOH functionally inhibited NF-κB translocation in MG6 cells.

### 2.4. LPS-Induced Disappearance of IRAK1 in MG6 Cells and Its Reversal by GGOH

**Figure 5:**
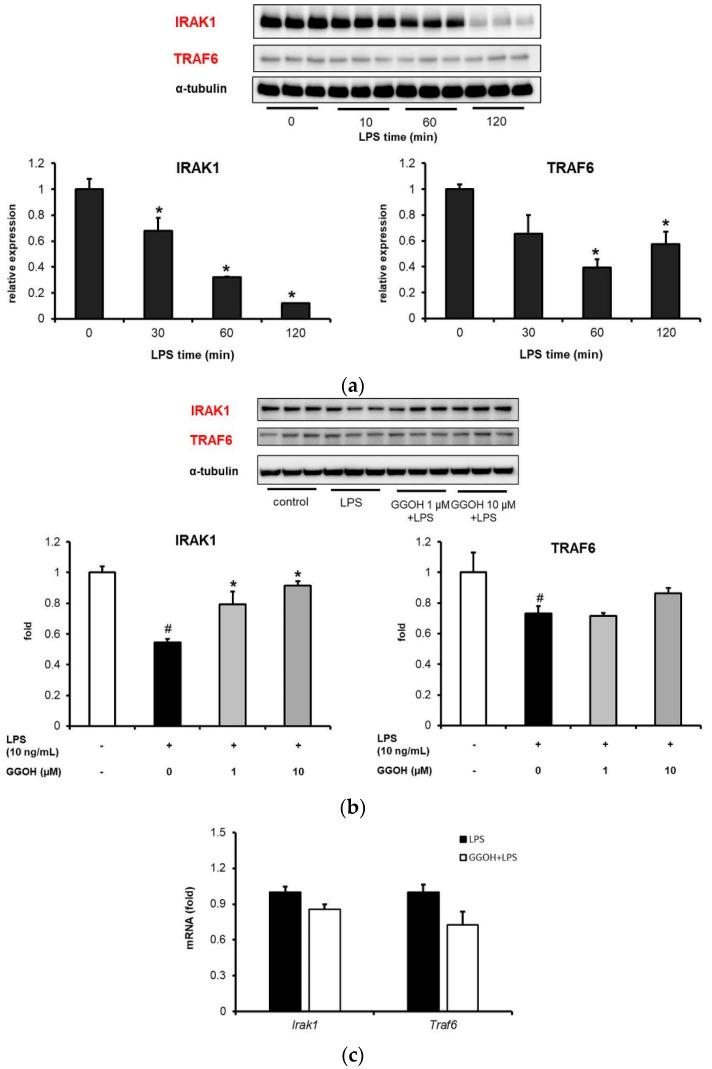
Reversal of LPS-induced NF-κB upstream protein expression by geranylgeraniol (GGOH) administration. MG6 cells were pre-treated with GGOH for 24 h followed by LPS treatment (10 ng/mL) for 1 h. (**a**) Western blot images and quantifications of IRAK1 and TRAF6 expression levels after different LPS administration times. (**b**) Western blot images and quantifications of IRAK1 and TRAF6 expression levels in the presence of GGOH followed LPS treatment for 1 h. (**c**) *Irak1* and *Traf6* mRNA expression levels after 1 h of LPS treatment. Total cell lysates were collected and subjected to Western blot analysis or qRT-PCR. Data are presented as the mean ± S.E.M, n = 3, normalized to total protein levels detected based on α-tubulin or *Eef1a1* as internal standards. # *p* < 0.05 vs. untreated control; * *p* < 0.05 vs. LPS-only treatment or LPS in 0 min.

Upon LPS binding to TLR4, the recruitment of upstream regulators, including IRAK1, which interacts with TRAF6 and plays an important role in downstream NF-κB signaling occurs. Initially, the effect of a single LPS administration on the expression levels of IRAK1 and TRAF6 in MG6 cells was examined. As shown in Figure 5a, LPS treatment induced the disappearance of IRAK1 and TRAF6 bands in a time-dependent manner. Next, the evaluation of the effect of GGOH on this process after 1 h of LPS treatment showed that while LPS administration markedly reduced IRAK1 expression compared with that in the control group, GGOH pre-treatment significantly inhibited this effect (Figure 5b). However, TRAF6 expression, which was reduced by LPS administration, did not show any significant changes following GGOH pre-treatment (Figure 5b). An investigation to determine whether this situation was associated with the mRNA levels of both proteins revealed that GGOH pre-treatment did not change *Irak1* and *Traf6* mRNA expression levels compared with LPS treatment (Figure 5c). These results suggest that GGOH is involved upstream of NF-κB signaling in a transcriptionally-independent manner.

### 2.5. Attenuation of LPS-Induced Pro-Inflammatory Cytokine Expression by GGOH and Other Isoprenoid Analogues

**Figure 6:**
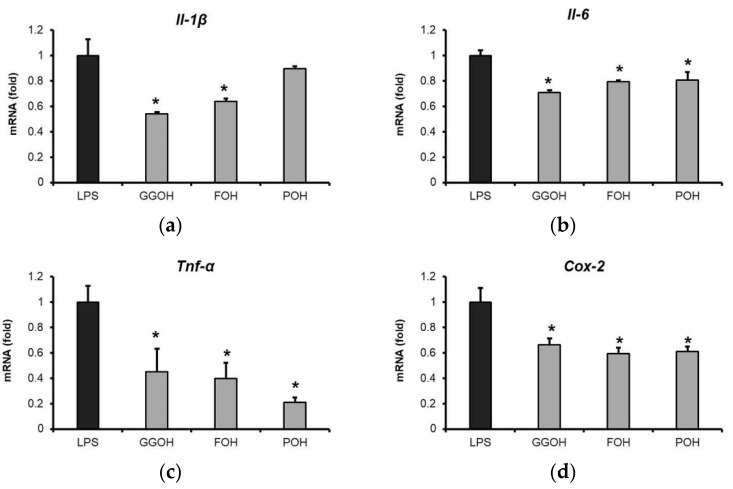
Suppression of pro-inflammatory cytokines mRNA upregulation by isoprenoid analogues. MG6 cells were administered isoprenoid analogues (10 µM) for 24 h followed by LPS treatment (10 ng/mL) for 3 h. (**a**–**d**) Pro-inflammatory cytokine mRNA expression following pre-treatment with isoprenoid analogues. mRNA levels were measured via qRT-PCR, normalized to the level of *Eef1a1* (the internal standard), and expressed as fold-changes relative to the LPS-only treated group. Data are presented as the mean ± S.E.M, n = 3; * *p* < 0.05 vs. LPS-only treatment group. GGOH, geranylgeraniol; FOH, farnesol; and POH, phytol.

A previous study showed that GGOH but not other isoprenoid analogs significantly control LPS-induced *Il-6* mRNA expression in THP-1 cells [36]. Therefore, in this study, an evaluation of the effects of isoprenoid analogues, GGOH, farnesol (FOH), and phytol (POH), on LPS-induced pro-inflammatory cytokine expression in MG6 microglial cells was performed. The results obtained showed that GGOH and FOH effectively downregulated the expression of pro-inflammatory cytokines in MG6 cells, while POH inhibited the expression of *Tnf-α*, *Il-6*, and *Cox-2*, but not *Il-1β* (Figure 6a–d). Apart from the cell-specific effect of GGOH, this finding demonstrated the potential positive effects of the administration isoprenoids, i.e., not only GGOH, but also other analogues, on LPS-induced pro-inflammatory cytokine expression.

### 2.6. Effect of GGOH Treatment on M2 Phenotype Polarization in MG6 Cells

**Figure 7:**
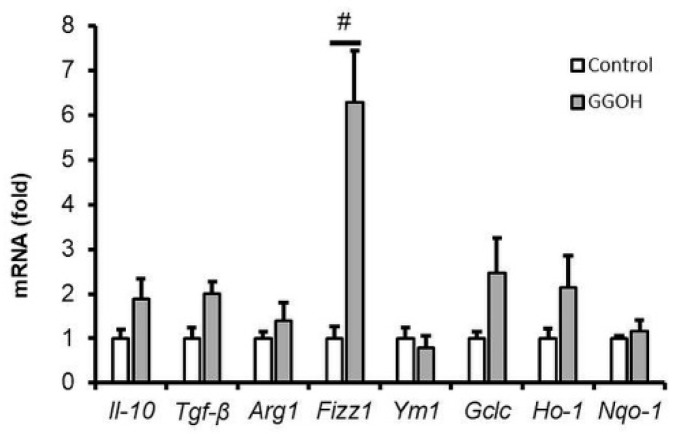
Non-regulation of the expression levels of M2 and Mox phenotype markers following geranylgeraniol (GGOH) administration. MG6 cells were pre-treated with or without GGOH (10 µM) for 24 h. Thereafter, mRNA expression levels were measured via qRT-PCR, normalized to the level of *Eef1a1* (the internal standard), and expressed as the fold-change relative to the values corresponding to the control cells. Data are presented as the mean ± S.E.M, n = 3; # *p* < 0.05 vs. control.

**Figure 8:**
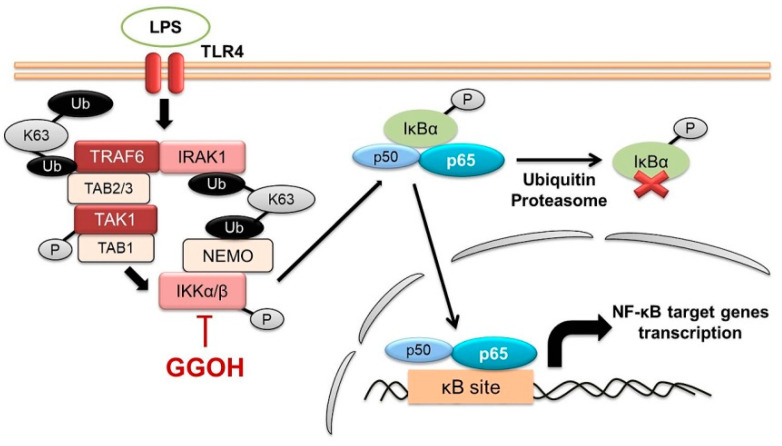
Possible molecular mechanism by which geranylgeraniol (GGOH) inhibited LPS-induced inflammation in MG6 cells. LPS induced NF-κB activation via the subsequent phosphorylation of TAK1, IKKα/β, p65, and IκBα, which triggered IκBα degradation and p65 nuclear translocation. However, GGOH administration inhibited IKKα/β phosphorylation, which possibly modulate the downstream signaling cascade. Additionally, it also reduced TAK1 phosphorylation and attenuated upstream signaling events, especially IRAK1. Even though further investigation is necessary, there is a possibility that GGOH interferes with the interaction between IRAK1 and NEMO, thereby reducing IKK phosphorylation effectivity.

As previously reported, microglia exist in two distinct phenotypes, namely, the M1 and M2 types, depending on the stimulus [3]. The M1 type represents pro-inflammatory phenotypes that generate inflammatory cytokines and reactive oxygen species (ROS), while the M2 type is characterized by the production of matrix-deposition or anti-inflammatory and wound-healing substances [7]. Figure 2 shows that LPS administration triggered M1 type polarization in MG6 cells by upregulating pro-inflammatory cytokine expression. To determine whether GGOH pre-treatment engendered the M2 type status in MG6 cells, we evaluated the expression of M2 markers by measuring their mRNA levels. As shown in Figure 7, we did not find any significant difference between the GGOH-pre-treated and the control groups with respect to the expression of M2 markers, including interleukin-10 (*Il-10*), transforming growth factor-β (*Tgf-β*), arginase 1 (*Arg1*), and *Ym-1*. However, it was observed that GGOH induced the expression of inflammatory zone 1 (*Fizz1*). Furthermore, we also analyzed the expression of markers corresponding to the Mox phenotype, which is a newly defined microglial activation state that is characterized by the upregulation of nuclear factor erythroid 2-related factor 2 (Nrf2) target genes [37]. Even though increasing tendencies were observed in this regard, there was no significant upregulation of the mRNA expression levels of glutamate-cysteine ligase catalytic subunit (*Gclc*), heme oxygenase 1 (*Ho-1*), or NAD(P)H quinone dehydrogenase 1 (*Nqo-1*) following GGOH administration (Figure 8).

## 3. Discussion

In this study, we demonstrated that GGOH pre-treatment effectively ameliorated LPS-induced inflammation in mouse-derived MG6 microglial cells. The anti-inflammatory action of GGOH was achieved via the inhibition of the NF-κB signaling pathway, as indicated by the lowering effects of this isoprenoid on the expression of pro-inflammatory markers. Additionally, FOH and POH administration also inhibited pro-inflammatory cytokine expression, and GGOH administration did not cause MG6 cells to show either the M2 or Mox phenotype.

It is well known that NF-κB signaling plays essential roles in the complex molecular mechanisms that regulate immune cell inflammation, including microglial cell inflammation. Additionally, NF-κB signaling induces the transcription of the pro-inflammatory cytokines that are responsible for chronic inflammation under perpetuating stimulation [38]. Previous studies have also shown that GGOH can minimize the expression of inflammatory markers in rat plasma and liver, human macrophage-like cells, and medulloblastoma cell lines [21,22,26]. Here, we showed that GGOH pre-treatment downregulated NF-κB target genes, including *Il-1β*, *Il-6*, *Tnf-α*, and *Cox-2* in MG6 cells. Thus, our results confirmed previous findings regarding the anti-inflammatory action of GGOH. The results also supported the hypothesis that GGOH might be beneficial in peripheral and CNS models. Furthermore, GGOH also effectively inhibited these inflammatory signals after a 24 h pre-incubation, while incubation for a shorter period only reduced *Tnf-α* mRNA levels. A similar pattern was observed in THP-1 macrophage-like cells [22]. Apart from the different synthesis kinetics of these genes, we expected that the anti-inflammatory properties of GGOH would have an indirect effect. This might be due to the conversion of GGOH into its metabolites or the production of anti-inflammatory compounds during the pre-incubation period.

By comparing the NF-κB p65 protein levels in the cytoplasmic and nuclear fractions and performing fluorescence imaging, it was clearly observed that GGOH inhibited NF-κB signaling in MG6 cells. Additionally, it was observed that the nuclear entry of NF-κB resulted from IκBα protein phosphorylation, which led IκBα to proteasomal degradation and NF-κB liberation. In this study, we also showed that GGOH administration inhibited IκBα phosphorylation induced by LPS while maintaining its integrity. Furthermore, it was unclear whether GGOH could directly affect the polyubiquitination of IκBα and 26S proteasome activity, given that our experiments did not involve ubiquitin–proteasome inhibitors. However, we observed that GGOH reduced the phosphorylation level of IKKα/β, which are the subunits of the kinase that propagates IκBα phosphorylation. Reportedly, the kinase activity of the IKK complex is attained after the phosphorylation of two serine residues in the activation loop of α/β subunits without any phosphorylation at the C terminus [39,40]. Therefore, the possible mechanism by which GGOH reduced IKKα/β phosphorylation was the interaction and oxidation of Cys-179 in IKKβ, as previously reported for other terpenoids [41].

Furthermore, our results also showed that GGOH inhibited TAK1 phosphorylation, which facilitated IKK complex phosphorylation. This prompted us to investigate the upstream regulators, including IRAK1 and TRAF6. Previously, it was considered that signaling via TLRs, such as LPS induces K48 polyubiquitination on IRAK1, leading to its degradation by the proteasome [42,43]. However, the results of another study showed that TLR-mediated ubiquitination of IRAK1 is realized by K63 instead of K48, which does not induce IRAK1 proteasomal degradation [44]. Additionally, K63-linked polyubiquitin mediates the binding of IRAK1 in the IRAK1–TRAF6 complex to NEMO, which is present as the IKK regulatory subunit and causes the unmodified IRAK1 band to be poorly detected. Conversely, K63 ubiquitination also facilitates TRAF6 and TAK1 linking via the TAB2/3 platform. This behavior might cause TAK1 and IKK colocalization to further phosphorylate the IKK complex [45].

The results of our experiments showed that LPS induced the IRAK1 band disappearance, while GGOH maintained its appearance. However, we did not observe any significant differences between LPS and GGOH with respect to the TRAF6 profile. Additionally, no significant differences were observed with respect to *Irak1* and *Traf6* mRNA levels, indicating that GGOH functioned in a transcriptionally independent manner. Although further investigations are urgently needed in this regard, there was a possibility that GGOH interfered with the K63–ubiquitin activity between IRAK1 and NEMO in LPS-induced inflammation in MG6 cells. Furthermore, we speculated that an interruption between IRAK1 and NEMO would affect the effectiveness of IKK phosphorylation. Nevertheless, our results suggested that GGOH probably attenuates upstream signaling, which inhibits further signal transduction.

In this study, we demonstrated that GGOH administration for 24 h did not effectively induce the expression of M2 phenotype markers in MG6 cells, except *Fizz1* expression. *Arg1* and *Fizz1* are the M2 markers that are important in inflammatory resolution and tissue repairs [46,47,48]. It was unclear whether GGOH only strongly induced *Fizz1* expression. Nevertheless, there were also increasing tendencies in other markers, although without statistical differences. How GGOH regulates M2 polarization warrants further study. GGOH also did not upregulate Nrf2 target genes (i.e., the Mox phenotype). In this regard, we surmised that the positive effect of GGOH was obtained preferably by its conversion into its derivatives rather than on the generation of anti-inflammatory factors within 24 h of pre-incubation, as mentioned above. Our speculation is also supported by a previous finding that NF-κB is inhibited by diterpenoid derivatives from geranylgeranyl pyrophosphate [49].

In general, we observed that GGOH, FOH, and POH exert a similar inhibitory effect on pro-inflammatory mRNA upregulation in MG6 cells. However, POH alone did not downregulate *Il-1β* mRNA expression. In previous studies, it has been considered that several factors affect the anti-inflammatory properties of isoprenoids, including their intracellular concentration, enzymatic interaction, phosphorylation to the pyrophosphate form, and association with other molecular pathways [23,50]. In this study, we believe that these isoprenoids can penetrate MG6 cells. However, whether they are associated with similar molecular pathways or not still requires clarification. Additionally, the results of this study extend our previous findings that GGOH, with a structure that is similar to that of the MK-4 side chain, exerts an effect on LPS-induced inflammation in MG6 cells that is a similar effect to that of MK-4 [32]. Therefore, it is likely that in addition to the naphthoquinone ring, the isoprene backbone also contributed to the anti-inflammatory effect of the isoprenoid. This outcome might reinforce the discovery of the potential of isoprenoids as NF-κB inhibitors [49].

To date, the pharmacokinetics of GGOH administration in humans has not been reported yet. There are also no reports citing GGOH concentration in the brain after its administration. In addition to these unknown facts, the function of exogenous GGOH has also not been sufficiently clarified. These notwithstanding, it has been reported that GGOH is required for hippocampal long-term potentiation, learning, and the rescuing of inflammatory markers in neuronal cells and animal models [19,20,21,26,31]. However, based on experiments involving cells, it has been reported that isoprenoids stimulate Aβ generation [51]. Further investigations have also suggested that the modification of Aβ is affected by changes in internal cholesterol levels rather than the concentration of isoprenoid products [52]. Moreover, isoprenoids exert harmful effects when they are administered at relatively high concentrations in non-neuronal cell lines [53]. Nonetheless, our data, which support the positive effects of GGOH, might facilitate the discovery of its new physiological aspects.

This study also had some limitations owing to the fact that it was based on a single cell-based experiment. Therefore, further analysis using human microglia as well as in vivo experiments are needed to confirm the current results and determine the optimum and safe dose. Additionally, in this experiment, we still limited our focus on the dynamic of NF-κB by GGOH. Thus, a wide-ranging proteomic analysis is also worth considering to intensify the understanding of GGOH molecular action.

Overall, this study collectively suggested that the attenuation of NF-κB signaling contributed to the anti-inflammatory action of GGOH on LPS-induced inflammation in MG6 cells, which is similar to the effect of MK-4. Therefore, these results represent a good starting point for further analysis to discover the function of GGOH and develop potential prodrugs that modulate inflammation in the CNS.

## 4. Materials and Methods

### 4.1. Materials

GGOH was obtained from Sigma-Aldrich (St. Louis, MO, USA). Farnesol (FOH) and phytol (POH) were purchased from Wako Pure Chemicals (Osaka, Japan). Furthermore, to obtain stock solutions at a concentration of 100 mM, these reagents were all completely dissolved in 99.5% ethanol. The final ethanol concentration in the cell medium was adjusted to 0.1% *v*/*v*. LPS (*Escherichia coli* serotype O111: B4) provided by Sigma-Aldrich was dissolved in phosphate buffer saline at 400 µg/mL and stored at −20 °C until further use.

### 4.2. Cell Cultures and Treatment 

The murine microglial cell line MG6 was obtained from RIKEN Cell Bank (Tsukuba, Japan) and Sigma-Aldrich, respectively [54,55]. These cell lines were cultured in a DMEM medium (Sigma-Aldrich) containing 10% fetal bovine serum (Biowest, Nualliè, France), penicillin (100 units/mL), and streptomycin (100 µg/mL). The MG6 cells were also supplemented with 100 µM β-mercaptoethanol (Wako Pure Chemicals) and 10 µg/mL human recombinant insulin (Gibco Thermo Fisher Scientific, Waltham, MA, USA). Furthermore, the cells were maintained in a humidified atmosphere incubator with 5% CO_2_ at 37 °C and passaged when they reached approximately 80% confluency. This was followed by the pre-treatment of the MG6 cells with GGOH for 24 h, which was followed by treatment with LPS (10 ng/mL) for 3 h, unless otherwise stated. 

### 4.3. Cell Viability Assay 

Cell viability was evaluated using water-soluble tetrazolium salt-1 (WST-1) assay. Specifically, the cells were seeded in a 96-well plate and administered with ethanol 0.1% (*v*/*v*) as the control and with GGOH at 0.1, 1, 10, and 100 µM for 24 h. After the incubation period, the WST-1 premix reagent (Takara Bio Inc., Shiga, Japan) was added, and absorbance indicating cell viability was measured at 450 nm, with a reference wavelength of 630 nm.

### 4.4. RNA Extraction and Quantitative RT-PCR Assay

Total RNA was isolated from MG6 cells using ISOGEN reagent (Nippon Gene, Tokyo, Japan) according to the manufacturer’s protocol. Then, the quantity of RNA was measured by determining the absorbance at 260 nm. Four micrograms of RNA were used to synthesize cDNA by denaturing them with oligo-dT primers and dNTPs at 65 °C for 5 min in a TaKaRa PCR Thermal Cycler Dice system (Takara Bio). Then, the mixture was incubated again with an RT buffer mixture containing 50 mM Tris-HCl (pH 8.3), RNaseOUT RNase inhibitor, and SuperScript III reverse transcriptase for 60 min at 50 °C, which was followed by 15 min incubation at 70 °C. Quantitative RT-PCR was performed using a CFX Connect Real-Time PCR Detection System (Bio-Rad, Hercules, CA, USA). The cDNA aliquot was amplified using the gene-specific primers listed in Table 1 and the TB Green Premix Ex Taq solution (Takara Bio). The expression level of each gene was normalized to the eukaryotic elongation factor 1α1 (*Eef1a1*) gene.

### 4.5. Immunoblot Analysis

To prepare whole-cell extracts, MG6 cells were lysed in cold buffer containing 50 mM Tris-HCl (pH 7.5), 5 mM EDTA, 150 mM NaCl, 0.1% SDS, and 1% NP-40. The lysis buffer was supplemented with proteinase and phosphatase inhibitors (Roche Applied Science, Mannheim, Germany). Thereafter, the protein amount was determined using the Lowry method and denatured in SDS gel loading buffer. Equal amounts of proteins were separated using 10–20% SDS-polyacrylamide gel (Wako Pure Chemicals) and transferred via a semi-dry transfer cell (Bio-Rad) into a PVDF membrane (Millipore, Billerica, MA, USA). After blocking with TBS-T containing 3% bovine serum albumin (Sigma-Aldrich), the membranes were incubated overnight at 4 °C with the following primary antibodies: anti-phospho-NF-κB p65 (Ser536), anti-NF-κB p65, anti-phospho-IκBα (Ser32), anti-IκBα, anti-phospho-IKKα/β (Ser176/180), anti-IKKβ, anti-phospho-TAK1 (Thr184/187), anti-TAK1, and anti-IRAK1 all from Cell Signaling Technology (Danvers, MA, USA), as well as anti-TRAF6 from Funakoshi (Tokyo, Japan). Furthermore, the protein bands were visualized using Immobilon Western Detection Reagent (Millipore) using the ChemiDoc Imaging System (Bio-Rad, Richmond, CA, USA), and densitometric analysis was performed using Image Lab 6.1 (Bio-Rad) normalized to α-tubulin (Sigma-Aldrich) or anti-lamin (Cell Signaling Technology) as the loading control.

### 4.6. Cytoplasmic-Nuclear Fractionation

MG6 cells were first lysed in a buffer containing 320 mM sucrose, 3 mM CaCl_2_, 0.1 mM EDTA, 1 mM dithiothreitol (DTT), 2 mM MgCl_2_, and 0.5% NP-40 in the presence of proteinase and phosphatase inhibitors. After 20 min, the mixtures were centrifuged at 600× *g* for 15 min. Thereafter, the supernatants were immediately transferred to a pre-chilled tube as the cytoplasmic fraction. Conversely, the pellets were washed three times with cytoplasmic buffer without NP-40, and after washing, they were mixed with nuclear lysis buffer containing 20 mM HEPES (pH 7.9), 1.5 mM MgCl_2_, 0.2 mM EDTA, 420 mM NaCl, 1 mM DTT, 0.3% NP-40, and 25% glycerol. Then, the mixtures were incubated in ice and vortexed for 15 s every 10 min for 40 min. Finally, the mixtures were centrifuged at 13,000× *g* for 10 min. Then, the supernatants were collected as the nuclear fractions and stored at −80 °C until further use.

### 4.7. Immunocytofluorescence

The treated MG6 cells were fixed in 4% formaldehyde/PBS for 15 min and permeabilized for 5 min using 0.25% Triton X-100/PBS. Thereafter, the samples were blocked with 4% fetal bovine serum (Gibco) for 1 h. This was followed by incubation overnight at 4 °C with p65 primary antibody (Cell Signaling Technology), followed by secondary antibody incubation using Alexa Fluor 555 (Invitrogen, Waltham, MA, USA) at 25 °C for 1 h. Then, nuclear staining was performed using 1 µg/mL Hoechst 33258. Finally, the samples were visualized under a fluorescence microscope (Olympus IX81; Olympus, Tokyo, Japan).

### 4.8. Statistical Analysis

Statistical analysis was performed using SigmaPlot v12.5 (San Jose, CA, USA). After the normality test, the data were analyzed using one-way analysis of variance followed by multiple comparison tests using the Dunnett or Tukey–Kramer test. Student’s *t*-test was used to compare two independent groups. Statistical significance was set at *p* < 0.05.

## Figures and Tables

**Table 1:** Oligonucleotide sequences of the primers used in quantitative RT-PCR.

Gene	Forward Primer	Reverse Primer
*Il-1β*	CTGTGTCTTTCCCGTGGACC	CAGCTCATATGGGTCCGACA
*Tnf-α*	GACGTGGAACTGGCAGAAGAG	TCTGGAAGCCCCCCATCT
*Il-6*	AGAGGAGACTTCACAGAGGATACC	AATCAGAATTGCCATTGCACAAC
*Cox-2*	TGAGTACCGCAAACGCTTCT	CAGCCATTTCCTTCTCTCCTGT
*Irak1*	GCCAGCCAAAGAACTTGATAGAA	TACTCTGCTTGCCTTGCTCACA
*Traf6*	GGAATCACTTGGCACGACACTT	GGACGCAAAGCAAGGTTAACAT
*Agr1*	CAGAAGAATGGAAGAGTC	CAGATATGCAGGGAGTCA
*Fizz1*	CTGATGAGACCATAGAGATTATCGTG	GCACAGGCAGTTGCAAGTATCTCC
*Gclc*	GGCTCTCTGCACCATCAC	TCTGACACGTAGCCTCGG
*Ho-1*	CCTTCCCGAACATCGACAGCC	GCAGCTCCTCAAACAGCTCAA
*Nqo-1*	TTCTGTGGCTTCCAGGTCTT	AGGCTGCTTGGAGCAAAATA
*Ym-1*	AGAAGGGAGTTTCAAACCTGGT	GTCTTGCTCATGTGTGTAAGTGA
*Il-10*	TGAATTCCCTGGGTGAGAAGCTGA	TGGCCTTGTAGACACCTTGGTCTT
*Tgf-β*	TAAAGAGGTCACCCGCGTGCTAAT	ACTGCTTCCCGAATGTCTGACGTA
*Eef1a1*	GATGGCCCCAAATTCTTGAAG	GGACCATGTCAACAATTGCAG

## Data Availability

Data are contained within the article.
